# From dietary exposure to systemic toxicity: organ-on-chip and multi-organ microphysiological systems for green food toxicology

**DOI:** 10.3389/fnut.2026.1940509

**Published:** 2026-09-15

**Authors:** Wenhan Liu, Yi Lu, Na Xu

**Affiliations:** 1 Department of Laboratory Medicine, Shengjing Hospital of China Medical University, Shenyang, China; 2 Liaoning Clinical Research Center for Laboratory Medicine, Shenyang, China; 3 Department of Human Genetics, Radboud University Medical Center, Nijmegen, Netherlands; 4 Department of Translational Genomics, Faculty of Health, Medicine and Life Sciences, GROW Institute for Oncology and Reproduction, Maastricht University, Maastricht, Netherlands

**Keywords:** dietary exposure, food safety, green food toxicology, microphysiological systems, new approach methodologies, organ-on-chip, quantitative *in vitro*-to-*in vivo* extrapolation, risk assessment

## Abstract

Food safety assessment must protect diverse human populations from chronic, low-dose, and mixed dietary exposures, yet conventional animal and static cell models often incompletely represent human digestion, metabolism, microbiota, and vulnerable life stages. This critical review maps living Organ-on-chip (OoC) and multi-organ microphysiological system (MPS) studies published through 10 July 2026 that address food contaminants, additives, foodborne toxins or pathogens, particles, and diet-relevant environmental chemicals. Direct food-toxicology evidence was distinguished from route-adjacent studies, transferable validation, and governance evidence, and studies were appraised for exposure realism, organ connectivity, dosimetry, and regulatory readiness. Gut chips demonstrate flow- and route-dependent responses to mycotoxins, dioxins, metals, enterotoxins, and food-relevant particles; liver and placenta models resolve metabolism- and life-stage-specific effects; and gut–liver systems show how translocation can produce distant-organ injury. However, most studies still use purified single agents, short exposures, transformed cell lines, and nominal concentrations, whereas authentic food matrices, digestion, microbiota, chronic mixtures, human benchmarks, and interlaboratory transfer remain uncommon. We therefore define Green Food Toxicology through five auditable dimensions—human relevance, animal independence, exposure-led evidence, resource sustainability, and regulatory utility—and recommend qualifying OoC/MPS for narrowly defined contexts of use within integrated weight-of-evidence assessment rather than treating them as universal animal replacements.

## 1 Introduction

Food toxicology is asked to make unusually consequential inferences from incomplete evidence. It must address chemicals that may occur at low concentrations for decades, co-exposures that vary with diet and geography, complex matrices that modify bioaccessibility, microbial transformations that differ among individuals, and susceptible windows such as pregnancy and childhood. At the same time, the conventional evidence base often relies on high-dose animal studies and reductionist cell cultures. Animal studies remain valuable for integrated physiology, but interspecies differences in intestinal structure, transporters, xenobiotic metabolism, immune signaling, microbiota and life-course kinetics can weaken direct translation to humans. Static monocultures offer experimental control, but they generally omit flow, mechanical forces, tissue interfaces, circulating immune cells and organ-to-organ communication. The resulting gap is not simply ethical; it is a measurement problem.

Organ-on-chip (OoC) technologies seek to narrow that gap by combining living cells with engineered microenvironments that reproduce selected tissue functions under controlled flow, geometry and, where relevant, mechanical deformation. The term Microphysiological System (MPS) is broader and includes microfluidic and non-microfluidic models intended to recapitulate organ-level physiology. In this review, an OoC requires living tissue in a controlled microsystem and a demonstrated organ-relevant function. A microfluidic sensor that merely detects a toxin is therefore a lab-on-chip, not an organ-on-chip; a static organoid is not automatically an OoC; and placing cells in a channel without functional qualification does not establish organ mimicry (1, 2). This boundary is essential in food science, where “chip” is also widely used for analytical detection platforms. For the purposes of this review, OoC is treated as a subset of MPS: it contains living cells or tissues within a miniaturized engineered device that imposes controlled perfusion, gradients, geometry and/or mechanical cues and is qualified by at least one organ-relevant function. MPS is the broader category of *in vitro* systems that reproduce selected aspects of tissue or organ physiology and may be microfluidic, perfused at a larger scale, or otherwise dynamically controlled. Static organoids remain organoids unless they are integrated into a controlled exposure system that enables defined transport, exposure and functional readouts; analytical chips without living tissue remain lab-on-chip devices.

The intestine is an obvious entry module for ingested hazards, but local intestinal injury is only one possible outcome. A food-associated chemical may be released during digestion, transformed by microbiota, transported across mucus and epithelium, metabolized or bioactivated in the liver, distributed in free or protein-bound form, and cleared by the kidney. It may then affect the heart, brain, immune system, endocrine tissues or the maternal–fetal interface. This causal sequence motivated early gut–liver and body-on-chip models for ingested substances (3, 4) and supports a modular “gut–X” architecture: the gut is coupled only to the downstream organ modules required by the toxicokinetic or mechanistic question. A multi-organ chip should thus reproduce a relevant exposure-to-outcome pathway, not attempt a decorative miniature of the entire human body.

Interest in food-relevant OoC has accelerated. The U.S. Food and Drug Administration (FDA) has explicitly evaluated liver-chip technology to understand chemicals in food, and the European Food Safety Authority (EFSA) has positioned New Approach Methodologies (NAMs) within future food and feed risk assessment (5, 6). Recent reviews have discussed food–microbiota–host interactions, nutrition in a One Health context, food nanotoxicology, biotoxins and organ-on-chip toxicology more generally (7–11). The literature nevertheless risks outrunning the evidence: statements that OoC “replaces animals,” “predicts human risk,” or is inherently sustainable are often made without specifying a regulatory decision, delivered dose, comparator or environmental footprint.

This review therefore makes four contributions. First, it separates direct food-toxicology applications from adjacent or transferable evidence rather than pooling all OoC studies. Second, it examines whether multi-organ connection changes interpretation relative to single-organ models. Third, it treats food exposure as a sequence—matrix, digestion, microbiota, absorption, metabolism and target-organ response—so that hazard identification is not mislabeled as risk assessment. Fourth, it operationalizes Green Food Toxicology (GFT) as a food-specific extension of established Green Toxicology, with auditable scientific, ethical, environmental and regulatory criteria. The central argument is deliberately restrained: OoC/MPS can reduce or replace animal use for defined contexts of use when their reliability and relevance are demonstrated, but current evidence does not support a universal one-to-one substitution.

## 2 Review methodology and evidence boundaries

### 2.1 Structured search strategy

A structured critical evidence map was developed rather than a statistical meta-analysis. Searches were updated to 10 July 2026 using PubMed-indexed records, publisher databases and citation chaining. Query blocks combined (“organ-on-chip” OR “organ chip” OR “microphysiological system” OR “body-on-chip” OR “multi-organ-on-chip” OR “gut-on-chip” OR “intestine-on-chip” OR “liver-on-chip” OR “placenta-on-chip”) with (“food” OR “food safety” OR “toxicology” OR “contaminant” OR “additive” OR “mycotoxin” OR “biotoxin” OR “pesticide” OR “dioxin” OR “metal” OR “microplastic” OR “nanomaterial” OR “enterotoxin” OR “foodborne” OR “nutrition”). Regulatory and methodological searches additionally targeted FDA, EFSA, OECD and the European Commission Joint Research Center (JRC).

PubMed was the bibliographic database queried with the fixed concept-block expression reported above; that expression is the complete PubMed search string used for the final update. Targeted searches of publisher-hosted journal platforms and backward and forward citation chaining were iterative and were used to identify route-adjacent, transfer and governance evidence. Because those supplementary searches were not logged as independent reproducible database searches, they are reported as supplementary identification routes rather than as additional systematic database searches.

Eligible primary studies contained a living OoC/MPS and evaluated at least one of the following: (i) a contaminant, additive, process-derived chemical, particle, toxin or pathogen relevant to food or dietary exposure; (ii) intestinal transport or bioavailability directly relevant to an ingested hazard; or (iii) organ crosstalk that materially informs food-toxicology design. Reviews, static organoids and drug studies were used only for context or transferable validation. Purely analytical lab-on-chip detection systems, non-living digestion devices without linkage to biological interpretation, and generic microfluidic cultures lacking organ-relevant qualification were excluded from the direct-evidence set.

Static organoids were not included as direct OoC/MPS evidence solely because they were three-dimensional or human-derived. An organoid-based study entered the direct-evidence set only when the organoid was integrated into a controlled microfluidic or perfused system, the food-relevant exposure was applied within that system, and an organ-relevant function or transport process was demonstrated. Static organoids, organoid-conditioned media and analytical devices without living tissue were retained only as contextual or transfer evidence.

### 2.2 Evidence tiers

Evidence was classified before interpretation:

- **Tier 1—direct food-toxicology evidence:** a living OoC/MPS is exposed to a food contaminant, food additive, foodborne toxin/pathogen, food-relevant particle or an authentic contaminated food.
- **Tier 2—route- or decision-adjacent evidence:** the agent can be diet-borne or the model addresses a relevant vulnerable population, but the experimental route or matrix is not an ingested food scenario.
- **Tier 3—transfer evidence:** drug, disease or nutrition studies validate a function needed for food safety, such as first-pass metabolism, chronic perfusion, microbiome co-culture, organ crosstalk or predictive performance.
- **Tier 4—governance evidence:** regulatory, standardization and good-method-practice documents define how an OoC result could become decision-relevant.

This hierarchy prevents two common category errors. A platform that successfully predicts drug-induced liver injury supports liver-chip credibility, but it does not by itself validate the platform for a food contaminant. Likewise, an intestinal barrier response to a purified chemical is hazard evidence, not a quantitative estimate of dietary risk unless external exposure, bioaccessibility, internal dose and population variability are linked.

### 2.3 Critical-appraisal domains

Each direct or adjacent study was examined across eight domains: biological source and identity; organ-relevant function; food and route realism; nominal vs. free or delivered dose; device-material interactions and mass balance; exposure duration and mixtures; comparator and benchmark strategy; and readiness for a defined decision. These domains were chosen because technical sophistication alone does not guarantee toxicological validity. A simple model with controlled dosimetry and a clear context of use may be more decision-worthy than a complex connected system with unknown compound loss and no benchmark.

The eight-domain appraisal was used to structure narrative judgments and identify applicability boundaries; it was not a validated study-level risk-of-bias instrument, and no numerical quality score was assigned.

### 2.4 Limitations of the review approach

This review is a critical narrative evidence map rather than a systematic review or meta-analysis. It did not use duplicate independent screening or a formal risk-of-bias instrument, and the descriptive counts in Figure 1 can include overlapping categories. The conclusions therefore emphasize evidence boundaries and defined contexts of use rather than prevalence estimates or pooled effect sizes. The absence of duplicate screening and formal risk-of-bias assessment may have increased selection and interpretation bias. We therefore avoid claims of exhaustive coverage, prevalence or pooled certainty, and the evidence tiers should be read as an interpretive map rather than a complete inventory.

**Figure 1:**
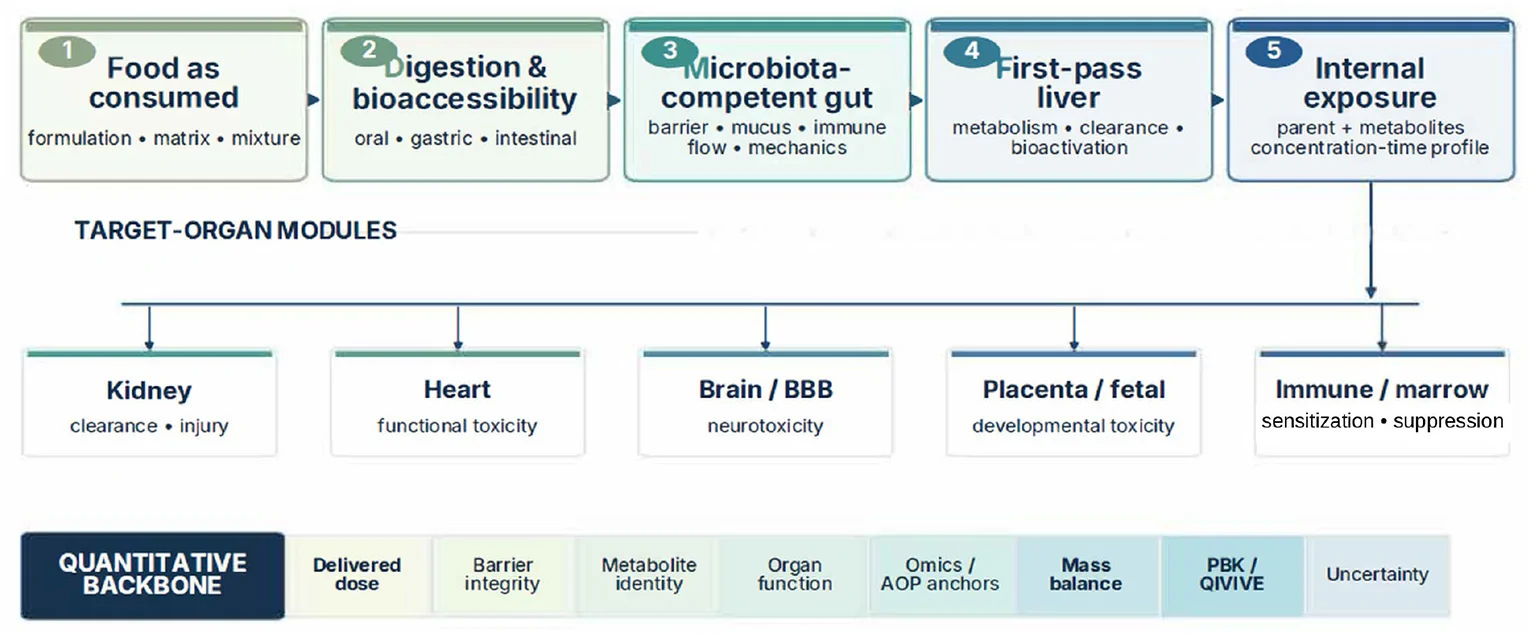
Evidence landscape for food-relevant organ-on-chip and multi-organ microphysiological-system studies. Cells show counts of representative study–platform applications summarized in Table 1, not effect sizes or quality scores; one study may contribute to more than one organ or hazard cell.

## 3 Why food exposure requires an axis, not an isolated organ

### 3.1 Food matrix and digestion define the test article

The toxicological test article is rarely identical to the chemical listed on a food label or analytical certificate. Gastric pH, digestive enzymes, bile, lipids, proteins, fiber and minerals can change dissolution, aggregation, binding and chemical form. These effects are especially important for particles, metals and lipophilic contaminants. Standardized INFOGEST methods improved reproducibility for static and semi-dynamic digestion, while digestion-on-chip systems introduced time-resolved flow and compartmentalization (12–15). However, coupling digesta to living intestinal tissue remains difficult because bile salts and digestive enzymes can injure cells, and the dilution or cleanup required for compatibility can distort the exposure that reaches the epithelium (16).

The evidence map exposes this bottleneck. Among direct food-OoC applications, the 2025 cadmium study using rice is a rare example in which a real food matrix was linked to a microfluidic intestinal model; measured bioavailability was lower than in a planar Caco-2 model and closer to a mouse comparator (17). Most other studies apply a purified agent in culture medium. Such experiments are valuable for mechanism and ranking but cannot resolve whether food processing or matrix binding amplifies or attenuates exposure. For regulatory use, authors should report the chain from concentration in food to concentration in digesta, concentration entering the chip, unbound concentration, tissue-associated mass and effluent recovery.

### 3.2 The gut is a dynamic barrier and a metabolic ecosystem

Flow and mechanical cues alter intestinal differentiation, barrier properties and transport. The foundational human gut chip developed villus-like morphology under flow and cyclic strain and supported microbial co-culture (18, 19). Subsequent systems incorporated primary biopsy-derived organoids, endothelial interfaces, immune cells, oxygen gradients and complex microbiota (20–22). These features are not ornamental. They can change the direction or magnitude of a toxicological response. In the deoxynivalenol (DON) study, barrier and inflammatory responses depended on application route and flow; in mercury transport, cyclic strain changed absorption and transporter expression; and in Shigella infection, tissue architecture and peristaltic forces increased invasion at lower inocula than static models revealed (23–25).

Microbiota add another layer of toxicokinetics and susceptibility. They can activate, detoxify or sequester xenobiotics, alter mucus and barrier integrity, and generate metabolites that act locally or systemically. Organ chips can maintain epithelial–microbial contact longer than conventional aerobic monoculture, but stability, representativeness and donor effects remain unresolved. Human colon chips identified microbiome metabolites that enhanced enterohemorrhagic Escherichia coli pathogenesis and helped explain species-specific susceptibility (26). This is strong evidence that human-specific microenvironmental context matters, yet it is not evidence that a generic “microbiome chip” predicts population risk. Food toxicology needs defined microbial communities, compositional and functional quality controls, metabolite mass balance, and explicit handling of interindividual variability.

#### 3.2.1 Microbiome–OoC integration requires controlled ecology

Microbiome integration is not simply the addition of a fecal inoculum to a gut channel. Many dominant colonic organisms require a low-redox, anaerobic lumen, whereas the epithelium and endothelial compartment require oxygenation. Platforms must therefore establish and verify a spatial oxygen gradient, control gas permeability and leakage, and report oxygen or redox measurements over time. Medium composition, fermentable substrate, pH, bile acids, flow, dilution, mucin supply, inoculum density and antibiotic carryover can each shift community structure or metabolite production; washout, overgrowth, biofilm formation and ecological drift may occur even when the epithelial barrier remains intact (7, 18, 22).

Representativeness requires both compositional and functional quality control. At minimum, studies should report inoculum source and processing, donor characteristics relevant to the question, community composition at baseline and during exposure, absolute microbial load, viability, key metabolite production and recovery, and predefined stability criteria. Defined consortia improve reproducibility and mechanistic attribution, whereas donor-derived communities better capture interindividual variation but require donor panels, replication at the donor level and hierarchical analysis. A single pooled inoculum should not be presented as population coverage.

A universal “microbiome chip” is therefore unlikely to be fit for all food-toxicology decisions. The microbial community, intestinal region, dietary substrate, exposure duration and downstream host module should be selected according to the causal question—for example, microbial bioactivation of a contaminant, detoxification of a foodborne toxin, barrier modification or production of a circulating metabolite. Generic platforms may provide an engineering chassis, but the biological model will remain context-specific. Validation should focus on the relevant microbial function and host consequence rather than taxonomic resemblance alone (26).

### 3.3 The liver converts exposure into a moving target

The liver may detoxify a compound, generate a more toxic metabolite or change its protein binding and distribution. Consequently, direct application of a parent compound to a distal organ can create the wrong hazard. Liver chips can maintain metabolic functions under perfusion and have shown predictive value in pharmaceutical toxicology; a blinded evaluation of a human liver chip distinguished several clinically hepatotoxic drugs from less toxic analogs, illustrating the type of performance study that food applications still lack (27). Food-specific studies are fewer. A rat liver-on-chip combined with transcriptomics and metabolomics resolved dose-dependent steatotic signatures after DDT, permethrin and their mixture (28). The rat-cell basis constrains direct extrapolation to human biology and therefore limits claims of direct human replacement. Conversely, the use of human-derived cells would not by itself establish human relevance; functional qualification and benchmarking against human data would still be required. HepaRG-based chips have examined aflatoxin B1 and emerging enniatins, demonstrating that metabolic competence and three-dimensional context change toxicity interpretation (29–31).

### 3.4 Distal organs and vulnerable life stages

Food-associated toxicants may affect organs that are not directly exposed to food. Kidney modules can address clearance and proximal-tubule injury; blood–brain barrier and neural modules can address neurotoxicity; cardiac tissues can measure electrophysiology and contractility; immune modules can capture activation or suppression; and placental/fetal-interface chips can model pregnancy-specific transport and signaling. The last category provides particularly relevant evidence that a tissue may respond even when chemical transfer is limited. A six-cell second-trimester placenta chip exposed to endocrine-disrupting chemicals showed cell-specific oxidative, endocrine and inflammatory effects, while a four-cell fetal–maternal-interface chip found that maternal exposure to BPA, PFOA, PBDE-47 and DDT could trigger fetal-membrane inflammatory signaling despite limited propagation to fetal chambers (32, 33). These systems do not yet reproduce gestational pharmacokinetics, endocrine feedback or long-term development, but they make a central point: vulnerable-life-stage toxicology cannot be inferred from an adult gut or liver module alone.

OoC/MPS may also address limitations of conventional animal models for vulnerable populations, but life-stage specificity must be designed rather than inferred. Pregnancy models should reflect gestational stage, maternal and fetal tissue identity, endocrine context and gestational PBK when available. Childhood models require developmentally appropriate intestinal, hepatic, renal, immune and neuroendocrine functions because transporter expression, metabolic capacity, barrier maturation, body-water distribution and protein-binding conditions differ from healthy adults. Adult-derived tissues exposed at lower concentrations are not automatically pediatric models. Donor-derived organoids and induced pluripotent stem-cell systems could support age- or genotype-specific panels, although their maturation state and benchmark requirements remain substantial (20, 32, 33).

Figure 2 summarizes the minimum sufficient exposure-led architecture proposed for food toxicology.

**Figure 2:**
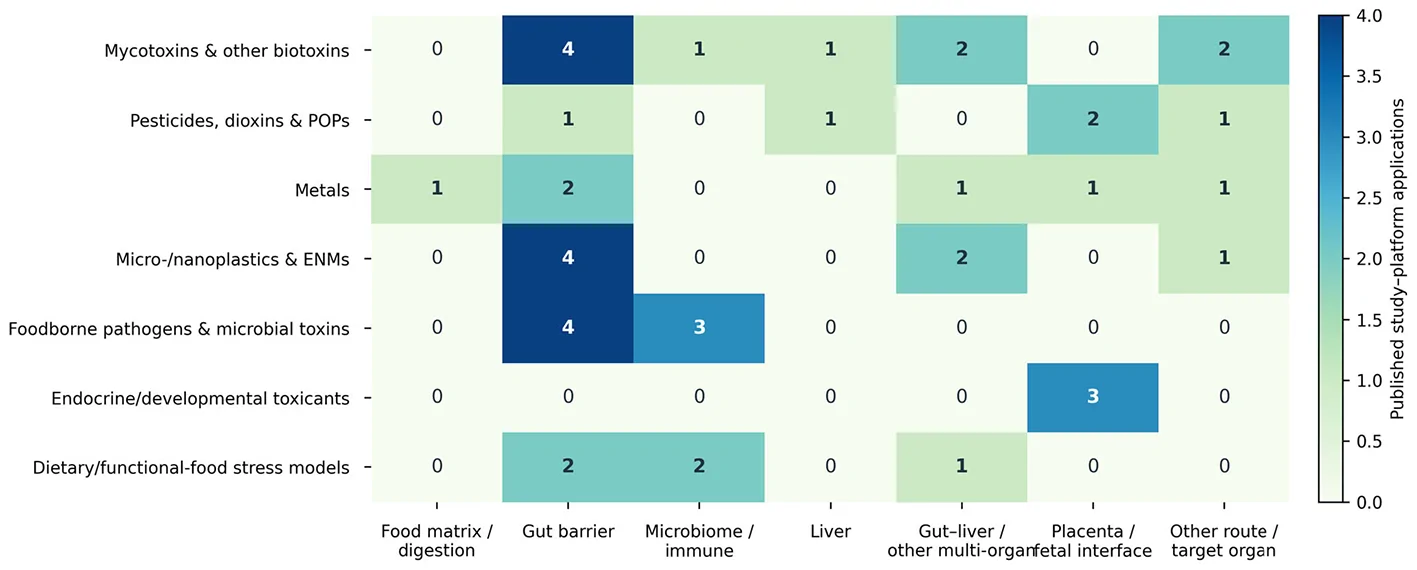
Exposure-led multi-organ architecture for food toxicology. A minimum sufficient chain begins with food as consumed, digestion, and a microbiota-competent gut barrier; downstream modules are added only when they change toxicokinetics or interpretation. BBB, blood–brain barrier; PBK, physiologically based kinetic; QIVIVE, quantitative *in vitro*-to-*in vivo* extrapolation.

## 4 What has actually been demonstrated?

The direct evidence base is small enough that its boundaries should remain visible. Table 1 summarizes representative primary studies and identifies what each establishes—and what it does not. The pattern is more informative than a simple publication count: mechanistic evidence exists across dioxins, pesticides, metals, mycotoxins, endocrine disruptors, particles and microbial hazards, but authentic foods, long-term low-dose exposure, complex mixtures and cross-laboratory qualification are rare.

**Table 1:** Representative direct and adjacent food-toxicology applications of living OoC/MPS.

References	Tier	Hazard/exposure	OoC/MPS design	Decision-relevant inference	Primary boundary	Regulatory relevance/near-term application
Imura et al. (3)	2	Ingested model substances	Intestine–liver micro total bioassay	Established a sequential absorption–metabolism–bioactivity design for ingested substances.	Transfer evidence; simplified cell lines and fluidic scaling.	Architectural transfer evidence for sequential absorption–metabolism assays; not yet decision-qualified.
Esch et al. (4)	1	Polystyrene nanoparticles	Gastrointestinal–liver body-on-chip	Connected exposure suggested downstream hepatic injury after gastrointestinal passage.	No food digestion; early platform and limited human benchmarking.	Mechanistic support for gut–liver linkage and prioritization of particle hazards.
Kulthong et al. (34)	1	Lipophilic dioxin congeners	Dynamic intestinal barrier chip	Demonstrated a perfused intestinal barrier for food-contaminant biokinetics.	No digestion, microbiome, liver or systemic target organ.	Relative intestinal transport and biokinetic screening for lipophilic contaminants.
Bovard et al. (29)	2	Aflatoxin B1	Lung–liver chip	Showed that a metabolically active liver module changed toxicity in the exposed barrier.	Inhalational, not ingestion; principle is transferable rather than food-specific validation.	Transfer evidence for testing metabolism-dependent hazard; route-specific qualification is required.
Grassart et al. (25)	1	Shigella flexneri	Human intestine-on-chip	Tissue architecture and mechanics increased invasion relative to static models.	Mechanistic infection model; lacks food-matrix survival and quantitative risk linkage.	Mechanistic pathogen–barrier studies and intervention ranking; not quantitative infection-risk estimation.
Tovaglieri et al. (26)	1	Enterohemorrhagic E. coli	Human colon chip	Identified microbiome metabolites that enhanced human-specific pathogenicity.	Does not represent population microbiome diversity or food-chain exposure.	Microbiome-mediated mechanism identification and strain or metabolite prioritization.
Schimek et al. (30)	2	Aflatoxin B1	Bronchial–liver multi-organ chip	Confirmed metabolism-dependent modulation across linked tissues.	Not an oral exposure chain and not a food-risk model.	Transfer evidence for metabolic modulation in connected systems.
Jellali et al. (28)	1	DDT, permethrin and mixture	Perfused liver-on-chip	Resolved compound- and mixture-specific metabolic and inflammatory signatures.	Rat-cell biology constrains direct extrapolation to humans; short exposure and mechanistic concentrations further limit the strength of human risk inference.	Mechanistic mixture profiling and biomarker discovery; not human risk estimation.
Kulthong et al. (38)	1	TiO2 and ZnO nanomaterials	Dynamic gut-on-chip vs. static culture	Flow-conditioned cells produced different molecular responses from static cultures.	No digestion or authentic food matrix; dynamic state complicates cross-model comparison.	Dynamic-vs.-static method comparison and mechanistic screening.
Donkers et al. (39)	1	Microplastics	Advanced lung and intestinal barriers	Demonstrated limited but measurable passage across advanced epithelial barriers.	Models were not a connected ingestion-to-organ system; particle dosimetry remains difficult.	Particle-translocation method development and comparative barrier ranking.
Koning et al. (42)	2	Nickel	Gingiva–skin multi-organ platform	Showed immune communication between a local exposure site and distant tissue.	Oral mucosa is not intestinal ingestion; exposure and disease context are specialized.	Mechanistic evidence for systemic immune signaling in route-adjacent exposure.
Kim et al. (37)	2	Cadmium	Feto-maternal interface chip	Provided life-stage-specific evidence for cadmium responses at the maternal–fetal interface.	Not linked to oral absorption or gestational PBK.	Developmental-mechanism support and prioritization for pregnancy-focused testing.
Pöschl et al. (23)	1	Deoxynivalenol	3D gut-on-chip with flow	Revealed dose-, route- and time-dependent barrier and inflammatory responses.	Purified toxin and short exposure; TEER direction alone was not diagnostic.	Barrier-liability ranking and route-sensitive dose–response characterization.
Wang et al. (24)	1	Hg(II)	Sensor-integrated gut-on-chip	Mechanical strain changed mercury transport and transporter-associated responses.	Purified metal species; no food matrix, speciation during digestion or human benchmark.	Transport and dosimetry method development with real-time barrier monitoring.
Delon et al. (40)	1	Model particles	Microphysiological intestine models	Developed quantitative methods to capture particle movement across intestinal tissue.	Model particles and assay-specific recovery limit direct food-risk interpretation.	Quantitative particle-recovery and transcytosis method qualification.
Morelli et al. (35)	1	Nigericin, patulin, ochratoxin A, melittin	High-throughput Caco-2 tubules	Distinguished toxin-specific multi-endpoint response patterns.	No digestion, microbiome or hepatic metabolism; transformed cell line.	Higher-throughput toxin classification and candidate prioritization.
Vidal et al. (32)	2	BPA, BPS and PBDE congeners	Six-cell placenta-on-chip	Resolved cell-specific effects in a complex developmental interface.	No oral exposure or whole-gestation kinetics; mechanistic rather than quantitative risk evidence.	Developmental-interface mechanistic evidence and endpoint selection.
Li et al. (17)	1	Cadmium in rice	3D villus-like microfluidic intestinal model	Estimated bioavailability closer to a mouse comparator than a planar Caco-2 monolayer.	Single matrix and platform; human predictive performance remains unqualified.	Relative bioavailability assessment of contaminants in food matrices.
Wang et al. (41)	1	Polystyrene microplastics	Gut–liver-on-chip with mechanics and flow	Peristaltic conditions reduced particle transfer and downstream liver stress.	Model particles, acute exposure and limited population benchmarking.	Mechanistic gut–liver linkage and prioritization of particle exposure conditions.
Moyer et al. (33)	2	DDT, BPA, PBDE-47 and PFOA	Four-cell fetal–maternal interface chip	Detected fetal-side signaling despite limited chemical propagation.	Experimental levels exceeded typical human blood concentrations; no oral or gestational PBK.	Mechanistic developmental signaling; supports hypothesis generation, not population-risk estimation.
Morelli & Queiroz (36)	1	Candidalysin	Gut-on-chip	Extended gut-chip toxicology to a fungal virulence peptide.	Closer to infection biology than conventional dietary contaminant assessment.	Mechanistic microbial-toxin screening and intervention ranking.
Coulet et al. (31)	1	Enniatin A1 and B1	3D HepaRG organ chip vs. 2D	Showed model-context differences for emerging Fusarium mycotoxins.	No digestion, intestinal transfer or chronic mixture; cell-line-specific applicability.	Comparative liver-hazard screening and model-selection evidence.

Tier 1, direct food-toxicology evidence; Tier 2, route- or decision-adjacent evidence. Table entries are critical summaries, not validation endorsements. The “Regulatory relevance/near-term application” column identifies a plausible role within an integrated evidence strategy; it does not indicate current stand-alone regulatory acceptance.

The distribution of these representative applications across hazard and organ categories is shown in Figure 1.

### 4.1 Persistent organic pollutants and pesticides

Kulthong and colleagues developed a dynamic intestinal barrier model specifically to study cellular association and transport of lipophilic dioxin congeners, establishing a clear early link between gut-on-chip technology and food contaminants (34). The work showed the value of a perfused barrier for biokinetics, but it did not reproduce digestion, enterohepatic cycling or systemic toxicity. Jellali et al. later coupled a rat liver chip with multi-omics to examine DDT, permethrin and their mixture. High exposures produced inflammatory, steatotic and metabolic signatures, and the mixture generated a broader molecular response than either compound alone (28). This illustrates how chips and omics can resolve mixture mechanisms, while also exposing three limitations common to the field: a non-human cell source that constrains direct extrapolation to human biology, short exposure, and concentrations chosen for mechanistic separation rather than dietary realism. The first limitation concerns species relevance; it does not imply that human origin alone is sufficient for validation, because functional qualification and benchmarking against human data are also required.

Placental and fetal-interface studies broaden the interpretation of persistent pollutants. Vidal et al. (32) used BPA, BPS and PBDE congeners in a human placenta chip, and Moyer et al. (33) examined DDT, BPA, PBDE-47 and PFOA across a fetal–maternal tissue topology. The systems captured localized, cell-specific responses that could be obscured in bulk cultures. Yet in the latter study, exposures exceeded typical human blood concentrations by one to two orders of magnitude. Such transparency is welcome: a mechanistic high-dose response can support biological plausibility, but it should not be reported as direct evidence of population risk. Accordingly, high-dose studies should be labeled as mechanistic or mode-of-action evidence unless dose–exposure translation and population variability are established.

### 4.2 Mycotoxins and other biotoxins

Mycotoxins are a compelling use case because they are food-borne, chemically diverse, often co-occur, and can target the gut, liver, immune system and nervous system. DON is currently the clearest intestinal example. In a three-dimensional perfused gut model, DON caused dose- and route-dependent barrier disruption and IL-8 release; lower exposures transiently increased rather than decreased electrical resistance, and basolateral exposure was more disruptive than apical exposure (23). These findings caution against equating a single TEER direction with toxicity and demonstrate why application geometry must be reported.

A high-throughput Caco-2 tubule platform compared nigericin, patulin, ochratoxin A and melittin. The agents produced distinct patterns of barrier loss, morphology and inflammatory response, supporting multi-endpoint classification rather than a generic viability readout (35). However, the platform used isolated toxins without food digestion or hepatic metabolism. A later gut-chip study of candidalysin demonstrated epithelial injury and inflammation from a fungal virulence peptide, extending utility to microbial biotoxins but remaining closer to infection biology than conventional dietary contaminant assessment (36).

Liver-containing systems reveal why organ linkage matters. In lung–liver chips, aflatoxin B1 toxicity in bronchial tissue was altered by the presence of metabolically active liver spheroids (29, 30). Although the exposure route was inhalational and therefore Tier 2 for food toxicology, the studies establish a transferable principle: a remote metabolic organ can reverse the apparent sensitivity of the exposed barrier. In 2026, a three-dimensional HepaRG organ-chip study compared enniatin A1 and B1 with two-dimensional cultures, directly addressing emerging Fusarium toxins (31). The next decisive step is not another single-toxin viability comparison; it is chronic, low-dose, food-relevant mixtures linked from digestion and gut transport to hepatic and immune outcomes.

### 4.3 Metals, particles, and nano-enabled food concerns

Metals and particles make dosimetry unavoidable. A sensor-integrated gut chip monitored Hg(II) transport and barrier damage in real time; mechanical strain changed absorption and expression of Piezo1 and DMT1, and the transport estimate differed markedly from a static Transwell (24). A microfluidic villus-like intestinal model applied to cadmium-contaminated rice estimated lower bioavailability than a planar Caco-2 model and values nearer to a mouse comparator (17). A fetal–maternal-interface chip has also been used to investigate cadmium signaling, demonstrating the value of life-stage-specific tissue topology (37). Together, these studies show that geometry and mechanics can materially change internal-dose estimates. They do not yet establish which model best predicts human dietary absorption across foods, nutritional status and chronic exposure.

Food-grade and incidental particles are another rapidly growing area. A body-on-chip study connecting gastrointestinal and liver tissues suggested that ingested polystyrene nanoparticles could produce liver injury not predicted from the gut module alone (4). Dynamic Caco-2 models later showed stronger transcriptomic responses to titanium dioxide and zinc oxide nanomaterials than static cultures, implicating flow-dependent cell state in hazard characterization (38). Other studies demonstrated passage of microplastic particles across advanced epithelial barriers and developed quantitative approaches for particle transcytosis (39, 40). Most recently, a gut–liver chip with peristaltic and hepatic flow conditions tracked polystyrene microplastic transport and associated hepatic oxidative injury; increased intestinal peristalsis reduced transfer and liver stress (41). This is among the strongest demonstrations of added value from multi-organ and biomechanical integration.

Nevertheless, particle studies face exceptional comparability problems: polymer identity, size distribution, morphology, surface chemistry, aging, corona formation, aggregation, fluorescent-label stability, particle number vs. mass, and contamination controls. Food nanotoxicology also requires digestion because pH, ions, bile and macromolecules alter particle state. An advanced model that receives pristine particles suspended in culture medium may be biologically sophisticated but exposure-poor. The appropriate comparison is therefore not “chip vs. animal” in the abstract, but whether the model measures a human-relevant delivered particle dose and the causal events required for a defined decision.

### 4.4 Foodborne pathogens and microbial toxins

Human intestinal chips have revealed mechanisms that are difficult to reproduce in rodents or static monolayers. Tissue microarchitecture and mechanical forces facilitated Shigella invasion (25); microbiome metabolites increased enterohemorrhagic E. coli pathogenicity in a human-specific manner (26); and high-throughput tubules differentiated enterotoxin injury patterns (35). These results are highly relevant to food safety, but risk assessment for biological hazards additionally requires inoculum dynamics, strain variation, food-matrix protection, gastric survival, microbiome competition, immune recruitment and clinically anchored dose–response. OoC may be particularly valuable for mechanism and intervention ranking, whereas quantitative infection risk will still require integration with exposure modeling, epidemiology and human outbreak data.

### 4.5 The central evidence gaps

Five gaps recur across hazard classes. First, the food is usually missing: purified compounds dominate, and only a few models incorporate real matrices or digesta. Second, exposure is usually acute: days-long culture capability is often used for model maturation, followed by hours of high-dose treatment. Third, nominal concentration substitutes for dosimetry: free concentration, tissue dose, device loss and recovery are incompletely characterized. Fourth, human relevance may be constrained by non-human cell sources or, in human-derived systems, inferred from cell origin rather than demonstrated by performance: Caco-2, HepG2 and HepaRG cells are useful but do not represent the diversity of healthy intestinal, hepatic or developmental physiology. Fifth, complexity is rarely benchmarked: connected systems are often compared with static monocultures but not with their own unconnected modules under matched flow, making the incremental value of organ crosstalk hard to isolate.

## 5 Discussion

Across the evidence base, the central question is not whether an OoC/MPS is more complex or merely human-derived, but whether it changes a decision-relevant inference relative to a simpler model. The following sections discuss when multi-organ coupling adds information, how Green Food Toxicology should be operationalized, what evidence is needed for qualification, and how current regulatory initiatives should be interpreted.

### 5.1 When does multi-organ connection add information?

The strongest justification for a multi-organ MPS is not resemblance to the human body; it is a causal question that cannot be answered by an isolated tissue. Connection is informative when at least one of four conditions applies. First, an upstream organ changes the identity, free fraction or concentration–time profile of the test article. Second, a downstream tissue clears or feeds back on an upstream signal. Third, an immune, endocrine or microbial mediator carries the response between organs. Fourth, benefit and harm must be considered simultaneously—for example, when a dietary intervention improves gut-barrier function but produces a metabolite with hepatic or cardiac liability. If none of these conditions applies, adding modules can increase variability, dilution and interpretive ambiguity without improving the decision.

#### 5.1.1 Metabolic competence can reverse the apparent hazard

The early gastrointestinal–liver systems of Imura et al. (3) and Esch et al. (4) showed why the order of exposure matters. In the latter study, gastrointestinal passage and liver exposure revealed nanoparticle-associated hepatic injury that was not reducible to the local gut response. Aflatoxin B1 lung–liver studies provide route-adjacent evidence in the opposite direction: hepatic metabolism altered the toxicity observed in the exposed bronchial tissue (29, 30). The recent gut–liver microplastic model extends this logic to an ingestion-relevant architecture and shows that intestinal mechanics can regulate both translocation and downstream liver stress (41). These studies collectively support a testable definition of “multi-organ added value”: the connected model changes a prespecified toxicokinetic quantity, mechanistic conclusion or hazard ranking relative to matched unconnected modules.

That definition requires an appropriate experiment. A convincing comparison includes: (i) each organ cultured alone under the same medium, flow and sampling schedule; (ii) connected modules; (iii) parent compound and relevant metabolites measured in inflow, tissue and outflow; and (iv) organ-function endpoints, not viability alone. A change that results merely from greater total cell mass, altered medium volume or nonspecific adsorption is a system artifact, not organ crosstalk. Few food-relevant studies currently make this full comparison.

#### 5.1.2 Immune and endocrine signals may travel without substantial chemical transfer

Physical passage of a parent chemical is not the only route to systemic effects. The gingiva–skin multi-organ model of nickel exposure demonstrated systemic immune activation across tissue modules, although the oral-mucosal route is adjacent rather than equivalent to ingestion (42). Placental and fetal-interface systems similarly show that local maternal-tissue stress can propagate inflammatory signaling even when transfer into fetal compartments is limited (32, 33). For food toxicology, this suggests a design priority: measure cytokines, endocrine mediators, extracellular vesicles and tissue function alongside chemical mass. Otherwise, a connected chip may miss a biologically important signal simply because the parent compound is absent downstream.

The same principle applies to the microbiome. A microbial metabolite produced upstream may be the true internal test article, yet its concentration can be highly sensitive to community composition, oxygen and substrate supply. Connecting a microbiota-competent gut module to liver, immune or cardiac tissue is therefore scientifically attractive, but only if microbial stability and metabolite recovery are demonstrated. Without those controls, the system cannot distinguish causal inter-organ signaling from day-to-day ecological drift.

#### 5.1.3 Scaling, recirculation, and common medium are biological assumptions

Multi-organ platforms cannot simply shrink human organs by the same geometric factor. Physiological scaling must preserve the quantities relevant to the question: surface area for absorption, metabolic capacity, tissue volume, perfusion, protein binding, residence time and secretory flux. Recirculating systems risk accumulating metabolites and depleting nutrients; open-flow systems can wash signals below detection. A common medium may improve engineering simplicity while compromising the specialized phenotype of one or more modules. The blood surrogate also lacks many *in vivo* buffering functions and can create unrealistic free fractions.

These limitations should be converted into model parameters rather than buried in the discussion. Investigators should report the liquid volumes of all reservoirs and channels, tissue cell numbers or functional capacities, flow paths, residence times, sampling losses, protein and lipid content, medium replacement, and the rationale for organ ratios. Physiologically based kinetic (PBK) modeling can then connect chip concentrations and fluxes to plausible human internal exposure. The goal is not anatomical miniaturization but quantitative similarity in the variables that control the selected outcome (43, 44).

#### 5.1.4 A modular gut–X strategy is more defensible than a universal body-on-chip

The evidence supports a modular architecture. The common upstream sequence is food as consumed → digestion/bioaccessibility → intestinal barrier, with microbiota and immune components included when they are causal. A liver module is added when first-pass metabolism, bioactivation, detoxification or biliary handling can alter the downstream dose. Only the target organ needed for the adverse outcome pathway is then connected: kidney for clearance and proximal-tubule injury; heart for electrophysiology or contractility; brain/blood–brain barrier for neurotoxicity; placenta/fetal tissues for developmental questions; or immune/marrow tissue for sensitization and suppression. This “gut–X” logic is technically tractable, hypothesis-led and compatible with staged qualification.

### 5.2 From organ chips to green food toxicology

Green Toxicology was developed before the present food-specific proposal as a strategy to identify and avoid toxic liabilities earlier and to align toxicology with sustainable chemistry (45, 46). Building on that foundation, we define Green Food Toxicology (GFT) as a food-safety paradigm structured around five auditable dimensions: human relevance, animal independence, exposure-led evidence, resource sustainability, and regulatory utility. GFT is not intended as a renaming of all non-animal food testing or as a claim of priority over the established Green Toxicology field.

These dimensions are specified as follows.

1. Human relevance. For claims of direct human replacement, appropriately characterized human cells or tissues are a necessary starting condition, but they are not sufficient on their own. The model must also reproduce the route and functions required by the question, include susceptible biology where relevant, and be benchmarked against human data.
2. Animal independence. The method reduces or replaces animal use within a stated context of use and progressively removes animal-derived serum, extracellular matrix, antibodies and reference tissues. “Animal-free” is reserved for systems with a documented animal-free supply chain.
3. Exposure-led evidence. The tested material reflects food as consumed, processing and digestion; delivered internal dose is measured; and *in vitro* effects are related to dietary exposure through PBK modeling and quantitative *in vitro*-to-*in vivo* extrapolation (QIVIVE).
4. Resource sustainability. Reagent volume, energy, water, device material, single-use plastic, hazardous waste, throughput, failed runs and carbon-intensive cold-chain requirements are measured against a relevant comparator. Small size is not accepted as proof of lower environmental impact. Operational metrics should use a defined functional unit and comparator and should include, where feasible, electricity (kWh), water and medium use (L), device and packaging mass, hazardous-waste mass, greenhouse-gas emissions, cold-chain burden, animal-derived inputs, valid results per week, data yield per sample and failed-run rate.
5. Regulatory utility. The method has a prespecified decision and acceptance criteria, transparent uncertainty, adequate controls, reproducible performance, version control and a route into weight-of-evidence assessment.

These dimensions and the minimum-claim rule are summarized in Figure 3.

**Figure 3:**
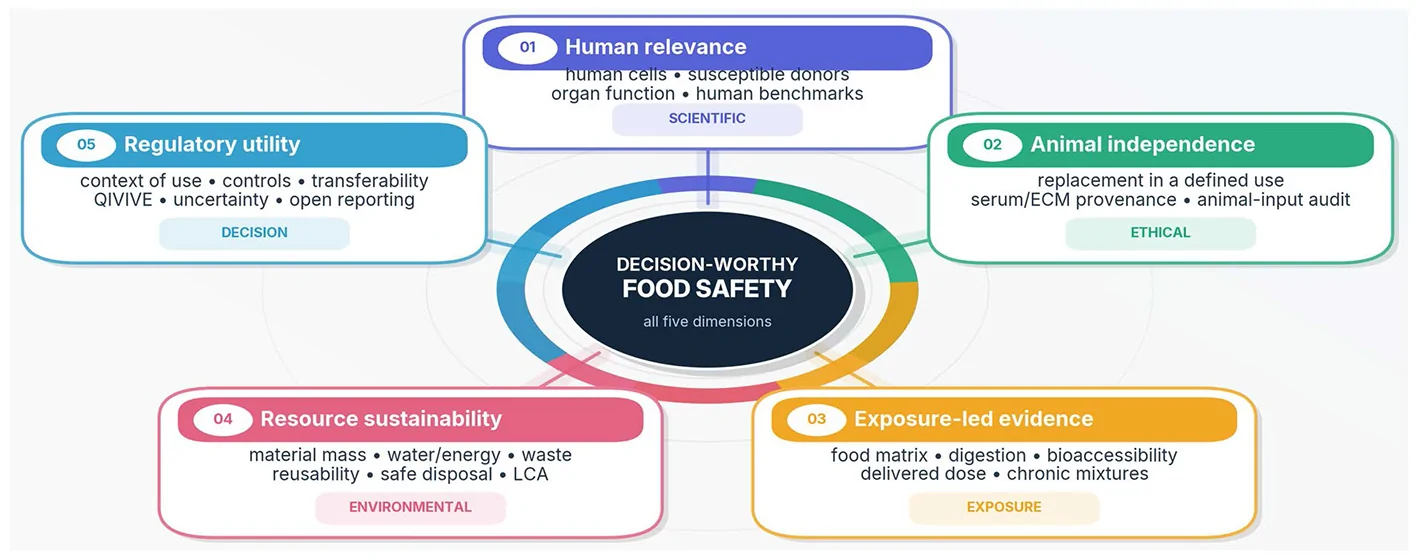
Operational framework for Green Food Toxicology. Five auditable dimensions integrate human relevance, animal independence, exposure-led evidence, resource sustainability, and regulatory utility.

This framework prevents “green” from becoming an ethical slogan detached from performance. A chip using human cells but large amounts of animal-derived matrix, single-use polymer and energy-intensive imaging may advance replacement while performing poorly on animal independence and resource sustainability. Conversely, a simple high-throughput barrier model may be greener and more useful than a multi-organ platform for initial prioritization, even if it is less physiologically elaborate. GFT therefore rewards minimum sufficient complexity: use the least resource-intensive model that captures the causal biology and achieves the required decision performance.

GFT is also a prospective design principle. Food developers could apply qualified MPS early to compare processing conditions, packaging migrants, ingredients or alternative proteins, selecting options with lower human-relevant hazard before scale-up. This “safe-and-sustainable-by-design” use is different from retrospective regulatory replacement: it can tolerate different uncertainty and may generate value before a method is accepted as a stand-alone regulatory test. Claims must nevertheless be proportional. A screening assay can rank candidates; it cannot establish population safety without exposure and uncertainty analysis.

### 5.3 Fit-for-purpose design, validation, and quantitative translation

#### 5.3.1 Start with the decision, not the device

Qualification begins with a context-of-use statement: the decision to be informed, chemical or particle domain, exposure route, population, biological endpoint, performance standard and role of the result. Plausible near-term contexts include ranking intestinal-barrier liabilities, identifying whether hepatic metabolism increases or decreases toxicity, estimating relative intestinal transfer, selecting compounds for further testing, or providing mechanistic support to an integrated approach. A claim such as “predicts food safety” is too broad to validate.

Four illustrative contexts show the required specificity. First, a perfused intestinal-barrier chip may rank packaging migrants or additives for delivered-dose-normalized barrier injury and permeability, with the result used to prioritize confirmatory testing. Second, a matched gut-only and gut–liver design may determine whether first-pass metabolism changes the hazard ranking of a mycotoxin or process contaminant. Third, a digestion–gut system may compare relative bioavailability from purified analyte, fortified food and naturally contaminated food. Fourth, a placental or fetal-interface chip may provide mechanistic evidence for developmental concern and select endpoints for an integrated assessment, but not by itself establish fetal population risk. For each example, the analyte domain, population, acceptance rule and role of the result must be prespecified.

The measurand must also be explicit. TEER is a convenient barrier metric, but it is sensitive to temperature, electrode geometry, cell density and transient tight-junction adaptation; it is not itself an adverse outcome. Viability can miss functional injury. Omics can reveal pathways but is vulnerable to batch effects and *post-hoc* interpretation. Each context of use therefore needs an endpoint panel spanning exposure, tissue integrity and organ function, with predefined rules for discordant results. Mechanistic anchors from an adverse outcome pathway can strengthen interpretation, but only when assay readouts are causally linked to the decision.

#### 5.3.2 Control the device before interpreting the biology

Microfluidic materials, tubing, seals and reservoirs can absorb hydrophobic chemicals, release uncured components, bind proteins or particles and alter oxygen transfer. Polydimethylsiloxane (PDMS) sorption is especially well documented (47). Flow and sampling can change the exposure faster than cellular uptake. Particle studies add sedimentation, aggregation, corona formation and label instability. Every study should therefore include cell-free recovery experiments across the complete fluidic path, time-resolved mass balance, solvent and device controls, and measurement of free or bioavailable concentration when protein binding matters.

Nominal concentration is an input instruction, not a dose metric. At minimum, reporting should distinguish concentration prepared, concentration entering the chip, time-weighted medium concentration, unbound fraction, tissue-associated amount, metabolite formation and effluent recovery. For particles, number, mass and surface area should be reported with size distribution and morphology before and after digestion or medium exposure. OECD guidance on nanomaterial preparation and dosimetry and the developing intestinal-fate guidance illustrate the level of characterization expected for decision use (48, 49).

For soluble chemicals, a minimum dosimetry dataset should include the concentration prepared; analytically measured inlet, outlet and reservoir concentrations at multiple time points; reservoir and channel volumes; flow rate and residence time; medium protein and lipid composition; unbound fraction; recovery from tubing and device materials; tissue-associated amount normalized to a defined biological denominator; metabolite concentrations; cumulative effluent mass; and overall mass balance. These data support delivered-dose metrics such as unbound Cmax, time-weighted concentration, area under the concentration–time curve, flux, clearance and time above a response threshold. Repeated-meal or pulse exposures require concentration–time data for each cycle rather than a single endpoint sample.

Dose–response analysis should therefore be based, where feasible, on delivered unbound concentration or tissue dose rather than nominal concentration. Effective concentrations, benchmark doses or other points of departure should be estimated with uncertainty and linked to the relevant exposure metric. Large divergence between nominal and effective dose may arise from protein binding, sorption, evaporation, degradation, metabolism, uptake or particle sedimentation; without time-resolved mass balance, an apparently steep or weak biological response may reflect exposure error rather than tissue sensitivity.

#### 5.3.3 Demonstrate biological identity and performance

Qualification should separate identity from function. Identity evidence includes cell source, donor variables, authentication, passage, differentiation, contamination control and the proportion of relevant cell types. Functional evidence includes barrier selectivity, transporter activity, metabolic competence, secretion, contractility, electrical activity or other organ-specific measures. Acceptance windows should be defined before toxicant exposure. A model that only displays marker expression is characterized, not validated.

Reference chemicals should span expected mechanisms and response ranges, include negatives, and be selected for the stated context rather than borrowed uncritically from pharmaceutical testing. Human benchmark data may include controlled exposure data, biomonitoring, clinical poisonings, epidemiology, explant responses and established toxicokinetics. No single source is perfect; triangulation is preferable to circular validation against an animal model the chip is intended to replace.

Positive controls should activate mechanisms and response ranges relevant to the stated food context rather than merely produce general cytotoxicity. True negatives are more difficult: a compound should be considered negative only within a defined route, matrix and delivered-dose range, with evidence that it reached the tissue and did not interfere with the assay. Poorly soluble or strongly sorbed compounds are not valid negatives when absence of response reflects absence of exposure. Benchmark sets should therefore include close structural or mechanistic analogs, matrix blanks, vehicle controls, assay-interference controls and, where possible, substances with contrasting human kinetic or clinical evidence.

Triangulation should test whether different human evidence streams converge despite their distinct limitations; it should not require exact agreement with any single dataset. The same animal dataset should not be used both to calibrate the chip and to claim independent validation, because that would create circular evidence.

#### 5.3.4 Reliability must be measured at several levels

Within-run repeatability, between-run reproducibility, donor and lot effects, operator effects, and interlaboratory transfer answer different questions. A platform can be repeatable within one laboratory yet fail when transferred because of proprietary media, pump calibration, imaging thresholds or tacit handling steps. OECD Good *In Vitro* Method Practices (GIVIMP) provide a relevant quality backbone: responsibilities, training, test-system definition, standard operating procedures, controls, equipment, data integrity, reporting and archiving (50). OoC-specific qualification additionally requires device manufacturing tolerances, leak and bubble controls, flow verification, sensor calibration and software/version traceability.

Table 2 consolidates the minimum evidence needed to qualify a food OoC/MPS for a defined decision.

**Table 2:** Minimum qualification evidence for a decision-ready food OoC/MPS.

Qualification domain	Minimum evidence	Frequent failure	Potential decision consequence
Context of use	Decision, chemical domain, population, endpoint and acceptance rule	Generic claim to “predict food safety”	A decision-relevant validation target may remain unclear.
Test article	Identity, purity, matrix, processing and digestion history	Purified analyte presented as food exposure	The applicability domain may be overstated.
Delivered dose	Inlet, free fraction, tissue amount, metabolites and effluent recovery; time-resolved inlet and outlet profiles, unbound Cmax and AUC, device recovery and overall mass balance	Nominal concentration only; no time-resolved mass balance	Potency estimates and QIVIVE may be unreliable. Dose–response estimates may reflect exposure error rather than tissue sensitivity.
Device control	Material recovery, flow, bubbles, leaks, evaporation and sensor calibration	No cell-free control or mass balance	Device artifacts may be misattributed to biology.
Biological identity	Cell provenance, authentication, donor variables, passage and contamination control	“Human-derived” used as sufficient validation	Human relevance may be assumed rather than demonstrated.
Organ function	Predefined barrier, metabolic, electrical, contractile or secretory acceptance windows	Markers or viability alone	The model may not adequately capture the target biology.
Comparators	Mechanistic positives, true negatives, matched static and unconnected modules	Only a conventional monoculture comparator	The added value of flow or connection may remain unresolved.
Reliability	Within-run, between-run, donor, operator and inter-site performance	Single laboratory and selected successful runs	Transferability and routine use may remain insufficiently supported.
Translation	Human benchmark, PBK/QIVIVE, uncertainty and prospective evaluation	Back-calculation from one nominal concentration	Hazard findings may be overinterpreted as risk estimates.
Change control	Versioned cells, media, device, software and bridging criteria	Unreported platform changes	Historical performance may not transfer after platform changes.
Operational feasibility	Cost per valid decision, hands-on time, skill level, throughput, automation, failure rate, instrument utilization and supply continuity	Only device price or best-case throughput reported without labor, analytics or repeat runs	Routine industrial feasibility and cost-effectiveness may be overstated.
Ethics and governance	Ethics approval or exemption, tissue provenance, consent scope, permitted secondary or commercial use, and privacy and data-access controls	Human-derived material treated as ethically self-validating	Legal, ethical or data-sharing acceptability may be compromised.
Sustainability	Animal inputs, energy, water, materials, waste, throughput and failure rate; electricity (kWh), water and medium (L), material and waste mass, greenhouse-gas emissions where feasible, and valid decisions per week	Small device assumed to be green	Environmental claims may remain insufficiently substantiated.

Formal validation does not always require a single centralized exercise before any use. A modular evidence strategy can qualify a platform for a narrow purpose, accumulate performance across laboratories and chemicals, and expand the context only when new evidence supports it. Changes to cells, matrix, device geometry, flow, medium or analytics require impact assessment and, when material, bridging studies. This is particularly important for commercial platforms whose components evolve.

Commercial implementation introduces an additional reproducibility problem: a platform name can remain constant while the chip polymer, geometry, membrane, coating, cell source, medium, pump firmware, image-analysis algorithm or recommended protocol changes. Publications should therefore report product and lot identifiers, hardware and software versions and the date-specific protocol used. Vendors should provide change notifications and bridging evidence, while users should retain vendor-independent controls and reference materials capable of detecting performance drift. Without this version control, historical validation cannot be assumed to transfer to an evolving proprietary system.

#### 5.3.5 PBK and QIVIVE convert chip responses into risk-relevant evidence

Hazard signals become risk-relevant only when connected to external exposure. A PBK/QIVIVE workflow uses the concentration–response relationship at the chip target site, corrects for binding and device losses, models human absorption, distribution, metabolism and excretion, and estimates the external intake associated with that internal response. Variability in age, pregnancy, disease, genetics, nutrition and co-exposure can then be propagated as distributions rather than hidden in a default uncertainty factor (51, 52).

Operationally, translation requires explicit scaling factors. Absorptive systems may be scaled by epithelial surface area and permeability–surface area product; metabolic modules by cell number, protein content, enzyme or transporter activity and intrinsic clearance; and connected systems by perfusate volume, flow, residence time and organ-capacity ratios. The choice should follow the governing process rather than uniform geometric miniaturization. Binding should be measured in the chip medium and, where relevant, converted to the human plasma or blood unbound fraction; total concentrations should not be compared when free fractions differ.

Time-resolved measurements are essential. Inlet, outlet, reservoir, tissue and metabolite concentrations should be sampled sufficiently to estimate Cmax, area under the curve, flux and clearance and to distinguish transient peaks from sustained exposure. These parameters, together with uncertainty in device losses and biological variability, can be propagated through PBK models to estimate external intakes or internal human concentration distributions. Calibration data, evaluation data and sensitivity analyses should be kept separate.

The chip contributes most when it supplies quantities that static cultures or animal extrapolation cannot estimate reliably: human intestinal permeability under flow, metabolite-specific production rates, barrier-to-blood flux, target-organ free concentration, and coupled functional response. Model calibration and evaluation must be separated, and uncertainty should include both PBK parameters and experimental variability. Back-calculating an intake from a single nominal *in vitro* concentration creates false precision.

#### 5.3.6 Adverse outcome pathways as a design and qualification backbone

Adverse Outcome Pathways (AOPs) can convert mechanistic ambition into a testable design. Starting from a defined adverse outcome and molecular initiating event, the relevant key events identify which tissue modules, exposure sequence and readouts are necessary and which are dispensable. For food OoC/MPS, an AOP-informed design may link metabolic bioactivation to hepatocellular stress, intestinal barrier disruption to inflammatory signaling, particle translocation to downstream oxidative injury, or endocrine perturbation to placental or fetal-interface responses. The chip need not reproduce the entire organism; it should reproduce the key events and relationships needed for the stated regulatory question (53, 54).

AOPs can also structure qualification. Reference chemicals should include substances expected to engage or not engage the molecular initiating event or key events; readouts should demonstrate temporal order, concentration–response concordance and biological plausibility; and connected modules should be justified only when they capture a causal key-event relationship. AOP mapping can support mode-of-action analysis, read-across and integrated approaches to testing and assessment, but it does not by itself quantify exposure or population risk and should not be treated as proof that all key-event relationships are complete.

#### 5.3.7 Economic and operational feasibility for routine use

Routine industrial adoption depends on total cost of ownership rather than the purchase price of a chip. Relevant costs include devices or plates, pumps and controllers, incubator and imaging capacity, sensors, primary or induced pluripotent stem-cell-derived tissues, proprietary media and matrices, analytical chemistry, PBK and data analysis, staff training and hands-on time, quality-control failures, maintenance, storage, waste handling and supply continuity. Cost per assay is uninterpretable unless the denominator is defined: one chip, one replicate, one chemical, one concentration series and one valid decision can yield very different estimates.

Feasibility studies should report cost per valid result or decision, valid runs per week, culture and assay duration, hands-on time, operator skill level, automation requirements, failure and repeat rates, instrument utilization and the number of chemicals that can be evaluated within the decision timeline. High-content and multi-organ systems may be justified for targeted questions but remain poorly suited to broad first-tier screening; simpler plate-compatible barrier models may provide greater throughput where organ crosstalk is not causal.

The existing liver-chip economic analysis illustrates how improved predictive performance could create value by avoiding costly downstream failures, but it was developed for pharmaceutical decision making and cannot be transferred directly to food-safety economics (27). Food-specific cost-effectiveness should compare OoC/MPS with the actual alternative workflow for the same decision, including animal studies, conventional *in vitro* assays, analytical testing and the cost of false-positive and false-negative decisions. Proprietary dependence, vendor lock-in and platform discontinuation should be included in sensitivity analyses.

#### 5.3.8 Ethics and governance of human-derived tissues

Greater human relevance does not eliminate ethical obligations. Studies using primary tissues, organoids or induced pluripotent stem cells should document ethics approval or exemption, provenance, the scope of informed consent, permitted secondary and commercial uses, and whether genomic or other potentially identifiable data are generated. De-identification does not remove all re-identification risk when donor genotypes, rare-disease status or linked omics data are shared. Governance should define access, data retention, withdrawal limits after cell-line derivation, incidental findings and recontact, and additional safeguards for tissues from children, pregnant donors or other vulnerable groups. Ethical and consent compatibility should therefore be part of model qualification and material-transfer due diligence, not assumed from the label “human-derived” (55).

Figure 4 presents the resulting layered qualification pathway.

**Figure 4:**
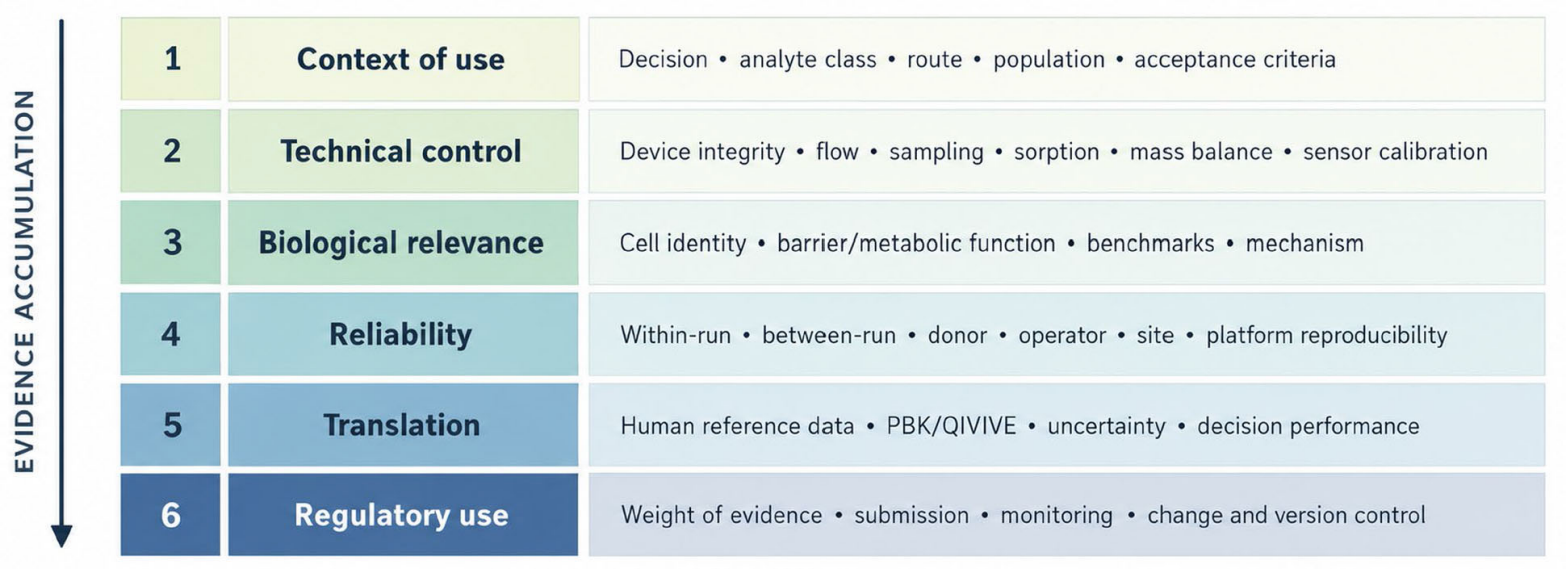
Layered qualification pathway for food organ-chip evidence. Evidence accumulates for a specific context of use; it does not create a universally “validated organ chip”.

### 5.4 Regulatory position: momentum without blanket acceptance

Regulatory interest is real, but the evidence does not support statements that food organ chips are already accepted as universal replacements. The FDA's Human Foods research program has evaluated liver-chip technology to better understand effects of chemicals in food on the human body. This demonstrates agency engagement, not the existence of a stand-alone regulatory requirement or acceptance criterion (5). EFSA's NAM roadmap places advanced *in vitro* systems within a broader program of exposure science, toxicokinetics, mechanistic evidence and weight-of-evidence integration (6, 56).

OECD frameworks are equally important because regulatory use depends on reproducible methods and interpretable data. GIVIMP supports laboratory practice; PBK guidance supports kinetic translation; omics reporting guidance supports transparent molecular evidence; and OECD's research-data guidance explicitly recognizes complex biological test systems (50, 57, 58). These documents do not confer automatic Mutual Acceptance of Data on an OoC assay. That generally requires an applicable OECD Test Guideline and Good Laboratory Practice, or acceptance by a regulator for a defined alternative route.

The JRC/EURL ECVAM has identified standardization as a prerequisite for OoC regulatory uptake, and international standardization efforts are addressing terminology, materials, fluidics, and performance (59). On 1 June 2026, the European Commission adopted a roadmap for phasing out animal testing in chemical safety assessment across multiple legislative domains, including food additives. The roadmap accelerates method development, validation, and uptake, but it is a staged transition rather than an immediate prohibition of animal data or an endorsement of every NAM (60).

Table 3 summarizes the resulting regulatory and standardization positions.

**Table 3:** Regulatory and standardization positions as of 10 July 2026.

Organization	Current position relevant to food OoC/MPS	What the position does not establish
U.S. FDA Human Foods Program (5)	FDA laboratories have evaluated liver-chip technology to understand effects of chemicals in food.	No universal stand-alone acceptance criterion for food organ chips.
EFSA (6, 56)	NAMs are being developed for food/feed assessment and are expected to contribute within structured weight of evidence and exposure-led risk assessment.	No general elimination of animal data or automatic acceptance of any platform.
OECD (48–51, 57, 58)	GIVIMP, PBK, omics, research-data and nanomaterial-dosimetry guidance provide quality and translation foundations for complex *in-vitro* systems.	OoC data do not automatically receive Mutual Acceptance of Data; there is no universal OoC Test Guideline.
JRC/EURL ECVAM (59)	Standardization, validation science and interlaboratory transfer are identified as prerequisites for regulatory uptake; multi-organ capabilities are under evaluation.	Laboratory capability or a roadmap is not regulatory endorsement of a method.
European Commission (60)	The roadmap adopted on 1 June 2026 covers multiple chemical-safety legislative domains, including food additives, and sets staged actions to replace animal testing.	The roadmap is not an immediate ban on animal studies and does not validate all NAMs.

The practical regulatory strategy is consequently plural. OoC evidence can already support problem formulation, mode-of-action analysis, read-across, prioritization, internal-dose estimation and the refinement of testing strategies. Greater decision weight becomes defensible when a method has a narrow context of use, controlled dosimetry, human benchmarks and transferable performance. Stand-alone replacement should be claimed only when the chip covers the decisive biology and achieves prospectively agreed performance; otherwise it belongs in an integrated approach to testing and assessment.

## 6 Future directions

### 6.1 Build benchmark sets around decisions

The field needs shared food-relevant benchmark sets rather than more isolated demonstrations. Each set should contain positive and negative reference substances, relevant metabolites, mixtures, and substances that expose known failure modes. Candidate decisions include intestinal permeability classification, metabolic bioactivation, developmental-interface signaling and particle translocation. Reference datasets should include human toxicokinetics and clinically or epidemiologically anchored effects wherever feasible. Blinded testing is essential to estimate predictive performance without analytical optimism.

### 6.2 Put food as consumed upstream of the chip

Matrix and processing should become experimental variables. Standard workflows are needed to connect INFOGEST digestion or dynamic digestion models to living gut tissues while preserving mass balance and cell viability. Studies should compare purified analyte, fortified food, naturally contaminated food and recovered digesta. Nutrient composition, cooking, fermentation, storage and packaging can then be studied as modifiers of bioaccessibility and toxicity, not treated as nuisance factors.

### 6.3 Replace acute high-dose demonstrations with realistic time courses

Chronic food exposure is often low, intermittent and mixed. Platforms must demonstrate stable phenotype and exposure control over weeks, or use repeated-pulse designs that reproduce meals and clearance. The scientifically relevant comparison is not simply acute vs. chronic culture but whether the concentration–time profile and accumulated tissue dose resemble humans. Mixture studies should test hypotheses grounded in co-occurrence and shared biology, using designs capable of distinguishing dose addition, interaction and kinetic competition.

### 6.4 Represent population variability deliberately

Primary and induced pluripotent stem-cell-derived tissues can capture genotype, sex, age, disease and nutritional state, but donor variation must be designed rather than sampled opportunistically. Reference donor panels, pooled vs. individual analysis, and hierarchical statistics are needed. Pregnancy, childhood, older age, metabolic disease and compromised barriers deserve priority because default healthy-adult models may not be protective. Microbiome variability should likewise be represented through defined communities and donor panels with functional quality controls.

### 6.5 Make the sustainability claim measurable

No general evidence yet proves that every OoC experiment has lower environmental impact than its comparator. Studies should report animal use displaced, animal-derived inputs retained, energy per result, reagent and water use, device and packaging mass, hazardous waste, failed-run rate, throughput and data yield. Comparative life-cycle assessment should use the same decision output as the functional unit—for example, “one correctly classified chemical within a defined domain,” not “one chip.” Reusable hardware, recyclable thermoplastics, serum-free media and right-sized analytics can then be optimized without sacrificing reliability.

### 6.6 Create an open, versioned evidence infrastructure

Data should be findable and reusable at the level needed for qualification: device geometry, materials, fluidic program, cell provenance, raw concentration data, endpoint definitions, code and deviations. A minimum information standard for food-OoC studies should align with GIVIMP, omics reporting and FAIR principles. Negative and technically failed studies are especially valuable because they define the applicability domain. Versioned reference materials and proficiency testing would support interlaboratory networks and prevent each platform from creating an incomparable private evidence base.

## 7 Conclusions

Organ-on-chip and multi-organ MPS have crossed an important threshold in food toxicology: they have moved beyond hypothetical promise to demonstrate flow-dependent intestinal transport, metabolism-dependent toxicity, particle translocation, life-stage-specific signaling and distant-organ injury. Yet the direct evidence remains sparse, heterogeneous and dominated by purified compounds, acute exposure and laboratory-specific platforms. The most persuasive studies are not those with the largest number of organs; they are those in which mechanics, metabolism or connection changes a measured internal dose or mechanistic conclusion.

Green Food Toxicology provides a disciplined way to develop the field. It joins human relevance and reduced animal dependence with authentic exposure, resource accounting and regulatory utility. Its governing rule is minimum sufficient complexity: reproduce the causal path from food as consumed to the decision-relevant outcome, measure the delivered dose, and add an organ only when it changes interpretation. Near-term regulatory value lies in defined components of integrated evidence—prioritization, mechanistic support, toxicokinetics and targeted replacement. Broader animal independence will require benchmarked performance, interlaboratory transfer, PBK/QIVIVE and transparent uncertainty. The transition is therefore not from “mouse to chip” in one step, but from poorly specified surrogates to qualified, human-relevant evidence systems.
